# Social Media Impact on Aesthetic Procedures Among Females in Riyadh, Saudi Arabia

**DOI:** 10.7759/cureus.6008

**Published:** 2019-10-28

**Authors:** Yazeed Alghonaim, Abdullah Arafat, Sarah Aldeghaither, Shaden Alsugheir, Salah Aldekhayel

**Affiliations:** 1 Otolaryngology, King Saud bin Abdulaziz University for Health Sciences, Riyadh, SAU; 2 Otolaryngology, King Abdulaziz Medical City, National Guard Health Affairs, Riyadh, SAU; 3 Otolaryngology, King Saud bin Abdulaziz University for Health Sciences, Riyadh, SAU; 4 Plastic Surgery, King Saud bin Abdulaziz University for Health Sciences, Riyadh, SAU

**Keywords:** aesthetic procedure, fascioplastic, saudi arabia, social media

## Abstract

Abstract

Aesthetic procedures represent one of the most commonly performed procedures in the medical field. Such procedures have been growing in popularity. Social media is a term used to describe electronic platforms that promote the dissemination of information to targeted users. These platforms play a critical role in promoting aesthetic procedures.

Objective

The objective of this study was to assess the impact of social media on aesthetic procedures among the female population in Riyadh, Saudi Arabia.

Methods

A cross-sectional study was performed. A validated online questionnaire, consisting of 26 questions, was distributed among females visiting the facial plastic clinic at King Abdulaziz Medical City, in Riyadh, Saudi Arabia.

Results

Out of 1449 participants, 81% were aged between 25 and 34 years. The majority (78.8%) were aware of the complications that may follow aesthetic procedures. The decision to undergo such procedures was affected by the price in 77%. Overall, 97% thought that cosmetic specialized accounts on social media are helpful, yet 77.8% believed that such accounts do not provide sufficient information. The influence of social media upon participants was 68%; 31.9% claimed that social media had no influence. Instagram was the most influential application followed by Snapchat and then Twitter; 50% of the participants routinely apply Snapchat filters and 42% decided to undergo facial changes after applying Snapchat filters.

Conclusion

Social media is an important source of information with Instagram being the most influential platform. Facial filters have led to an increase in the number of aesthetic procedures carried out.

## Introduction

Aesthetic procedures are considered to be one of the most commonly performed procedures in the medical field. These procedures have been growing in popularity in our nation as a result of multiple factors, including body image dissatisfaction and the pursuit of perfection, in addition to the growing diversity of social media and surgeon-related factors such as the surgeon’s reputation, board certification or years of experience. Social media is a term used to describe the electronic platforms which promote the dissemination of information to targeted users [1]. Moreover, social media plays a critical role in aesthetic procedures by providing visualized media to the target population. Historically, surgeons used their private web pages; however, nowadays modern surgeons are increasingly using Facebook, Instagram, Twitter, and other social media platforms. Such platforms have been used to improve communication, marketing, and education to colleagues and the public [2].

## Materials and methods

The Institutional Review Board of King Abdullah International Medical Research Center, Riyadh, KSA provided ethical approval for this study under protocol number RC18/178/R.

A cross-sectional study was conducted in Riyadh, Saudi Arabia between September 2017 and October 2018. Data was collected through an online questionnaire with explanatory cover for participation. The 26-questions questionnaire was validated using a face validity process through the judgment of experts and rating of non-expert rating through overt integrity tests. The questionnaire was then distributed among females visiting the facial plastic clinic at King Abdulaziz Medical City, in Riyadh, Saudi Arabia. All females above the age of 18 years living in Riyadh were included. The questionnaire was distributed with an explanatory letter requesting participation and written consent. 

The study was based on a structured questionnaire designed by the authors after extended research to better suit the research setting and environment. The questionnaire was available in two languages (Arabic and English). Data collection was performed using an online questionnaire to collect the data and was created using the Survey Monkey website. The questionnaire was assessed using Short Note on Statistical Package for the Social Sciences (SPSS), version 22.0 (IBM Corp, Armonk, NY) and by a Reliability Test. The questionnaire was used to collate data relating to demographic data (gender, age, nationality, occupation, and income) and participant knowledge (source of information, type of information, the reason for the procedure). We then evaluated the impact of social media on aesthetic procedures.

Statistical analysis

All collected data were analyzed using the SPSS package. Quantitative variables were evaluated using descriptive statistics, such as mean, standard deviation, and frequencies. All statistical analyses of qualitative data were performed using McNemar's test. Differences were considered to be significant when the P value was >0.05 and highly significant when the P value was >0.01.

## Results

A total of 1449 females, aged above 18 years and living in Riyadh, were included in our study. The majority of the participants (46.75%) were aged between 25 and 34 years; 55.09% were single. A high proportion of the study participants (77.29%) held a bachelor’s degree. The majority (37.79%) were employed, while 28.39% were unemployed and 25.82% were students. The monthly income of 47.41% of participants was below $1,333, 25.80% earned between $1,333- $2,666, and 13.39% earned $2,933- $4000. On the other hand, the monthly income of 8.15% and 3.97% were $4,266- $5,333 and $5,600-$13,333, respectively (Table 1).

**Table 1:** Demographics of the study

Age group	
Below 18 years / 18-24 years / 25-34 years / >35 years	32 (2%) / 440 (30%) / 677 (47%) / 300 (21%)
Marital status	
Single	921 (63%)
Married	528 (37%)
Level of Education	
Intermediate school	12 (.76%)
High School / Bachelor degree / Maters and doctorate / Others	160 (11%) / 1118 (77%) / 130 (9%) / 29 (2%)
Employment status	
Student	377 (26%)
Employed	552 (38%)
Unemployed / Retired / Self-employed	405 (28%) / 29 (2%) / 86 (6%)
Monthly income	
<5000 SAR	696 (48%)
5000-10000 SAR / 11000-15000 SAR / 16000-20000 SAR / 21000-50000	391 (27%) / 189(13%) / 116(8%) / 58 (4%)

Aesthetic procedure statistics

Of the respondents, 52% had previously undergone an aesthetic procedure, while 79% were willing to have an aesthetic procedure in the future. The majority (78.8%) were aware of the complications which may follow any aesthetic procedure (Table 2). The decision of having such procedures was affected by the price in 77% of participants. In terms of gender preference, 44.8% preferred a male doctor, while 6.3% preferred a female doctor; 34% had no gender preference.

**Table 2:** The decision of having aesthetic procedure

	Yes	No
Are you willing to have an aesthetic procedure?	1145 (79%)	304 (21%)
Have you ever had an aesthetic procedure?	696 (48%)	753 (52%)
Are you aware of the complication of aesthetic procedures?	1145 (79%)	304 (21%)
Does the price affect your decision about aesthetic procedures?	1130 (78%)	319(22%)

**Figure 1:**
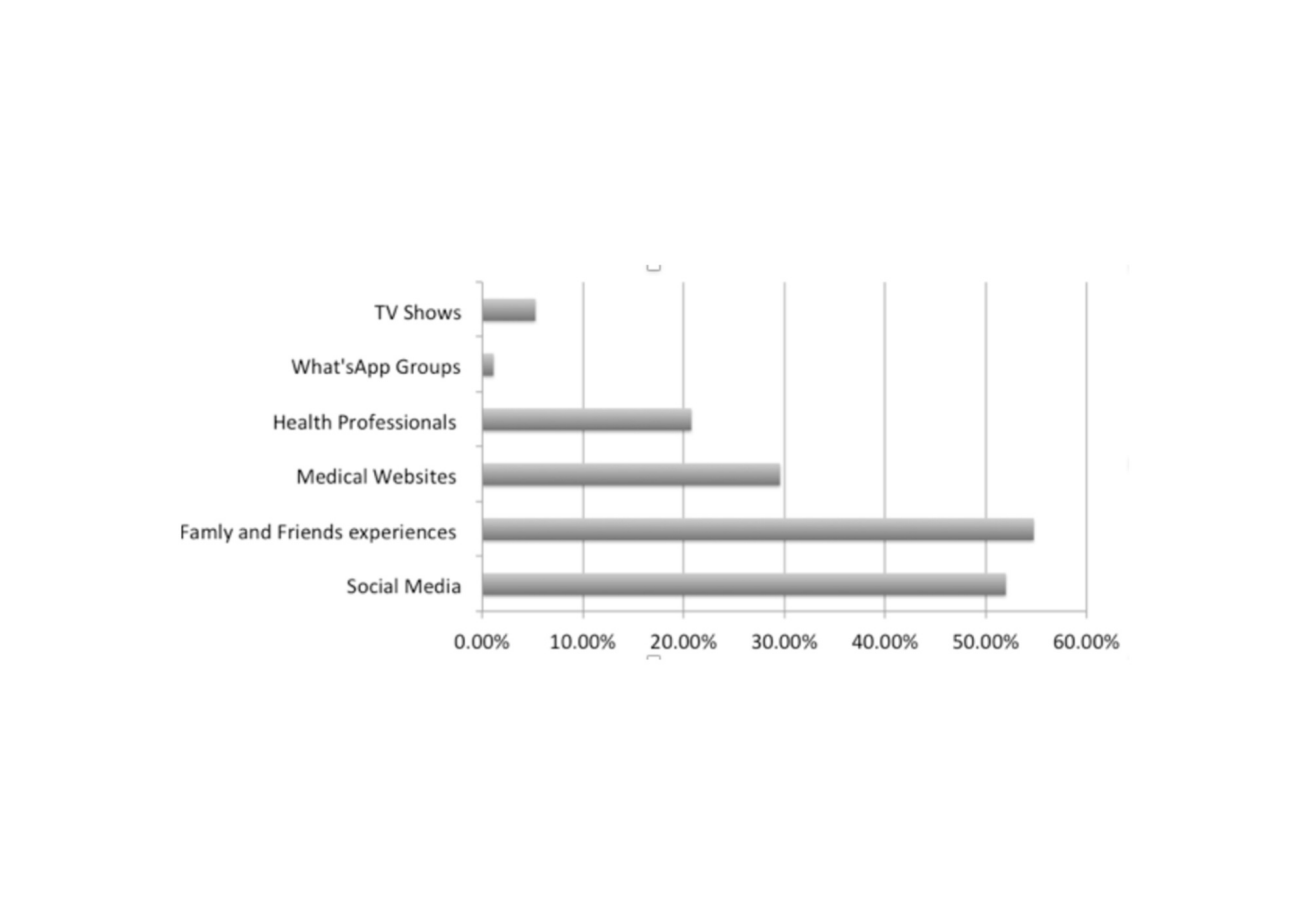
The best source of information for aesthetic procedures

**Figure 2:**
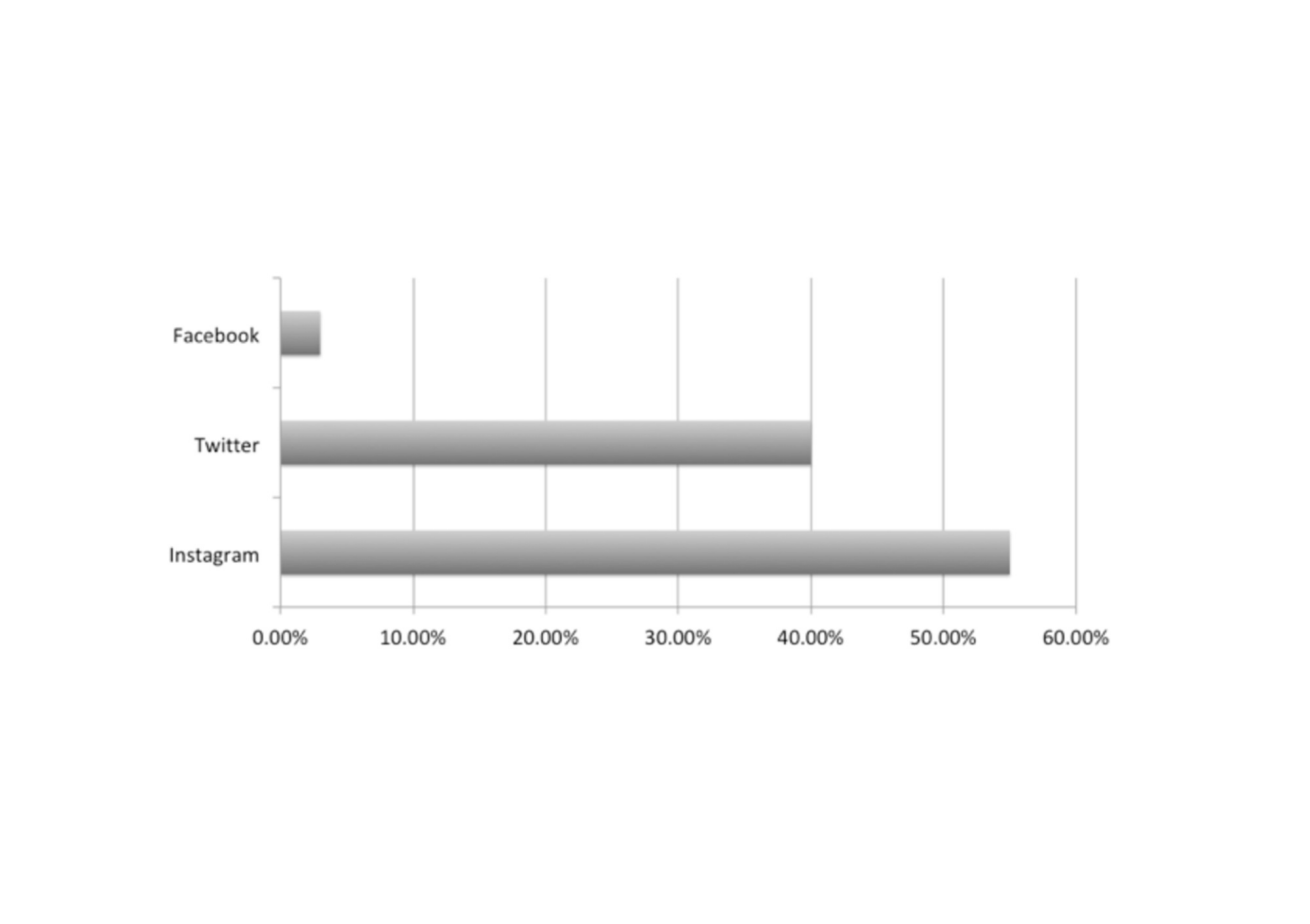
The most influential application

When asked to choose the best source of information for aesthetic procedures, participants were given the option of choosing more than one. Social media was chosen by 52% of participants while 55% chose the experience of family and friends, 30% chose medical websites, 21% chose health professions and 5% chose TV programs (Figure 1). Overall, 77.8% believed that social media accounts were not a sufficient source of information. Almost all of the participants (97%) reported that cosmetic specialized accounts on social media were helpful. Social media had a major influence upon participants to have such procedures, even if they did not need them in 21% of participants, had a minor influence on 47% and had no influence at all in 32%. The group of 25-34 year olds were the most influenced by social media (51%). Instagram was the most influential application (55%) followed by Snapchat (40%) and then Twitter (3%) (Figure 2). Furthermore, 50% of the participants always apply filters and 42% decided to have facial changes after applying Snapchat filters.

The experience of others, and opinions about the doctor, were common reasons underlying gender preference. Private hospitals were the most preferred location for aesthetic procedures; governmental hospitals were the least preferred.

There was a negative correlation between age and the desire to go for aesthetic procedure (r = -.036, n = 1441, p = .170). There was also a negative correlation between age and previous history of aesthetic procedure (r = -.156, n = 1441, p = .000) significant at the 0.01 level (2-tailed) and a negative correlation between age and complications awareness (r = -.026, n = 1432, p = .319) (Table 3). Furthermore, the Pearson’s (bivariate correlation) analysis showed a positive correlation between marital status and applying snapchat filters (r =. 101, n = 1430, p = .000) significant at the 0.01 level (2-tailed) (Table 4). There was also a positive correlation between marital status and did snapchat filters let you think of having some facial changes (r = .040, n = 1436, p = .134) (Table 4).

**Table 3:** Correlation between age, the desire to go for aesthetic procedure, previous history of aesthetic procedure, and complications awareness

Correlations						
		Age	Do you want to have an aesthetic procedure?	Have you ever had a cosmetic procedure?	Are you aware of the complications that might follow any aesthetic procedure?	
Age	Pearson Correlation	1	-.036	-.156^**^	-.026
	Sig. (2-tailed)		.170	.000	.319	
	N	1444	1441	1441	1432	
Do you want to have an aesthetic procedure?	Pearson Correlation	-.036	1	.241^**^	-.001
	Sig. (2-tailed)	.170		.000	.974	
	N	1441	1442	1439	1430	
Have you ever had an aesthetic procedure?	Pearson Correlation	-.156^**^	.241^**^	1	.178^**^
	Sig. (2-tailed)	.000	.000		.000	
	N	1441	1439	1442	1430	
Are you aware of the complications that might follow any aesthetic procedure?	Pearson Correlation	-.026	-.001	.178^**^	1
	Sig. (2-tailed)	.319	.974	.000		
	N	1432	1430	1430	1433	
**. Correlation is significant at the 0.01 level (2-tailed).						
*. Correlation is significant at the 0.05 level (2-tailed).						

**Table 4:** The Pearson’s (bivariate correlation) analysis

Marital status	If you are a Snapchat user, how often do you apply snapchat filters?	Did snapchat filters let you think of having some facial changes?
Marital status	Pearson Correlation	1	.101^**^	.040
	Sig. (2-tailed)		.000	.134
	N	1443	1430	1436
If you are a Snapchat user, how often do you apply snapchat filters?	Pearson Correlation	.101^**^	1	.299^**^
	Sig. (2-tailed)	.000		.000
	N	1430	1432	1429
Did snapchat filters let you think of having some facial changes?	Pearson Correlation	.040	.299^**^	1
	Sig. (2-tailed)	.134	.000	
	N	1436	1429	1438
**. Correlation is significant at the 0.01 level (2-tailed).				

## Discussion

Along with the power of the internet network, social media has become an important and fundamental part of many people's lives. This study showed that 20% of our participants believed that social media platforms had a negative influence on their lives, while 16% believed that they had a positive influence; 64% believed that they had both influences. This paper, involving a large study cohort, proved that Instagram was the most influential social media platform with regards to aesthetic procedures in Riyadh, Saudi Arabia. However, existing literature reports that Facebook is the most influential social media platform. A prospective study by Veld et al. (2017) found that 46% of patients had no gender preference, 26% requested a female surgeon and only 1% requested a male surgeon; the remaining 27% requested a specific surgeon due to her or his reputation [3]. Our present study showed that participants much preferred a male physician than a female physician.

In a previous cross-sectional study, Montemurro et al. (2015) distributed a questionnaire to 500 patients and 128 plastic surgeons to assess the impact of social media and the accessibility to online information relating to aesthetic procedures [4]. This previous survey found that 95% of participants used the internet to collect information prior to cosmetic consultation and 46% used social media specifically to gain information about the doctor and the procedure [4]. The results of our present study were slightly different as the majority of the participants were using social media, in addition to the experience of family and friends as their source of information for aesthetic procedures. Furthermore, our results showed that people aged 25-35 years were more willing to have aesthetic procedures compared to other age groups. We also found that Instagram was the most influential platform across all age groups. However, Facebook was used significantly more often in the group of participants who were aged over 35 years.

In another cross-sectional study, Chandawarkar et al. (2018) reviewed the qualifications of the top 100 social media influencers in plastic surgery in order to guide people as to whom to follow. The study showed that 77% of these influential people were plastic or facial plastic surgeons, 10% of other medical doctors and 13% were non-physicians [5]. In another study, Wheeler et al. evaluated the use of social media by 1000 aesthetic and plastic surgeons in marketing their practice; results showed that 28.2% used social media in their practice while 46.7% used it in their personal life only. Data also showed that most plastic surgeons used other ways to advertise their practice [6]. In a study aimed to create guidelines to use social media for clinical practice in an appropriate way, Chandawarkar et al. (2018) evaluated the content of Instagram accounts created by plastic surgery residents and found that 40 out of 67 accounts were active and posted photographs from their practice; only one of account posted images of patients [2]. The study also showed that there was a linear correlation between the number of posts and the number of followers [2].

Wong et al. (2011) compared the prevalence of classic marketing methods and social media in plastic surgery; results showed that plastic surgeons preferred social media platforms to enhance their visibility to potential consumers and connect with them. Furthermore, the number of patients utilizing the internet was significant and rising [7].

Gould et al. (2016) described social media as the most powerful tool for marketing used by physicians since it allows direct interaction between physicians and patients, as well as patients between each other. Gould et al. went on to state that social media can be used to educate patients, enhance marketing, and to exchange experiences [8]. In another study, Quinlan et al. (2016) assessed the use of social media platforms by plastic surgery journals and compared it with other specialties; results showed that the most common specialty using social media was plastic surgery. Moreover, 10 out of 24 journals had at least one social media account. Ten plastic surgery journals had Twitter accounts with a median of 525 followers per account. Twitter was the most commonly used platform, while Facebook, YouTube, Google+, and LinkedIn were used less commonly [9]. Margolin et al. (2013) described social media as a link between the surgeon and the patients; these authors believed that physicians could advertise and “brand” themselves through social media [10]. Humphries et al. (2016) discussed the potential role of social media in creating the brand of an academic plastic surgeon and found that social media was a place used to educate patients and to increase their knowledge of different conditions in aesthetic procedures. Moreover, patients used social media to find excellent physicians, to communicate with them regarding aesthetic procedures, and to discuss outcomes, complications, and prices [11].

## Conclusions

Our analysis found that social media represents an important source of information for aesthetic procedures, with Instagram being the most influential platform used by females living in Riyadh, Saudi Arabia. Moreover, the number of aesthetic procedures performed has risen because of the use of facial filters on social media. Finally, in order to provide comprehensive and valid information about cosmetic procedures, we suggest that physicians should create professional and scientific social media accounts.
